# Visceral Fat-Reducing Effect of Novel Dietary Intervention Program: A Randomized Controlled Trial in Japanese Males

**DOI:** 10.3390/nu16183202

**Published:** 2024-09-22

**Authors:** Naohisa Shobako, Hiroyasu Shimada, Tsuyoshi Yamato, Takuo Nakazeko, Yukio Hirano, Futoshi Nakamura, Keiko Honda

**Affiliations:** 1 Future Food Research & Development Division, Nissin Foods Holdings Co., Ltd., Tokyo 192-0001, Japan; 2 EP Mediate Co., Ltd., Tokyo 162-0821, Japan; 3 Kouwa Clinic, Tokyo 170-0003, Japan; 4 Laboratory of Medicine Nutrition, Kagawa Nutrition University, Saitama 350-0214, Japan

**Keywords:** visceral fat, obesity, randomized controlled trial, dietary reference intake, Optimized Nutri-Dense Meals

## Abstract

**Background/Objectives:** Reducing visceral fat is a critical factor in preventing obesity-related health complications. In this study, we evaluated the effectiveness of our previously reported novel dietary intervention program, “Optimized Nutri-Dense Meals”, designed according to the Dietary Reference Intakes (DRIs) in Japan in reducing visceral fat. **Method:** This open-label, randomized controlled trial included 100 Japanese males with obesity or overweight. In total, 50 participants were assigned to a control group that continued their usual diets, and the other 50 consumed test meals twice per week for four weeks (test group). **Result:** The primary outcome, visceral fat area, significantly decreased in the test group compared to that in the control group (−7.5 cm^2^, confidence interval [CI]: −14.3 to −0.6). In addition, we measured the changes in gut flora and work productivity. The abundance of *Bifidobacterium* (+1.5%, CI: 0.3–2.7) and *Christensenellaceae* (+0.4%, CI: 0.01–0.8) increased significantly in the test group compared with those in the control group. Work Limitation Questionnaire Japanese version scores representing presenteeism also significantly increased in the test group (+1.2 points, CI: 0.2–2.3) compared with the control group. **Conclusions:** These findings suggest that dietary interventions based on Japanese DRIs can effectively decrease visceral fat and improve health outcomes over the short term without the need for a complete dietary overhaul.

## 1. Introduction

Excess visceral fat is associated with multiple diseases, such as type 2 diabetes mellitus (T2DM) and cardiovascular disease (CVD) [1]. It is also a well-known independent risk factor of CVD morbidity and mortality. According to a previous systematic review, Asian people accumulate abdominal visceral fat more readily than Western people [2]. Thus, the reduction in excess visceral fat is essential among the Asian population.

Nutritional education and dietary interventions are the simplest and most well-studied methods to reduce visceral fat. Nutritional education seems effective in raising awareness about dietary issues; however, its positive effects on biomarkers are limited [3,4]. Daily diet is as important as exercise for preventing obesity and reducing visceral fat. However, a dietary intervention that can be easily adopted by anyone has yet to be discovered. Dietary interventions can be divided into calorie restriction and meal replacement. Calorie restriction has been reported to reduce lipid accumulation, oxidative stress, and inflammation in animal models of obesity [5], and a visceral fat-reducing effect has been observed in human clinical studies [6]. Although calorie restriction has demonstrated strong potential, Hemmingsson reported difficulty in continuously implementing this method, especially in relatively young and non-obese individuals [7]. However, the effectiveness of meal replacement has been well documented. In particular, traditional diets, such as the Mediterranean diet (MD) and traditional Japanese diet (washoku: “wa” means Japanese and “shoku” means meals or eating), have been researched. The MD is recognized as an intangible part of the cultural heritage of France, Italy, Greece, Spain, and Morocco by the United Nations Educational, Scientific, and Cultural Organization [8]. MD is characterized by a high intake of vegetables, legumes, fresh fruits, non-refined cereals, nuts, and olive oil, especially extra virgin oil; moderate intake of red wine; and low consumption of red meat [9,10]. The MD and washoku have been reported to exert anti-obesity effects [11,12]. Although these local traditional diets have attracted attention because of their reliable effects, continuous consumption of specific traditional foods for a month is difficult owing to the limited availability of ingredients and differing areas of residence [13]. Furthermore, previous dietary interventions have required full meal replacements during the program period.

Recently, we proposed the concept of Optimized Nutri-Dense Meals. The details of this concept have been described in a previous study [13]; briefly, we adjusted the nutritional content of 33 types of nutrients, including energy-producing nutrients. The amounts of these nutrients were confirmed to be within the reference range. Their criteria were based on the Dietary Reference Intakes (DRIs) in Japan. The most important feature of the Optimized Nutri-Dense Meal is the lack of a restriction on the menu as long as nutrient content is within standards. This idea contrasts with the MD, in which ingredients are specified in detail, such as olive oils and whole grains [14]. Thus, the Optimized Nutri-Dense Meals can be applied to various cultures’ menus, unlike the MD and washoku. In previous single-arm studies, the potential effects of reducing visceral fat area, modifying lipid metabolism and gut flora, and enhancing work productivity have been observed [15]. Therefore, Optimized Nutri-Dense Meals are expected to be a novel dietary intervention meal replacement that compensates for the weaknesses of traditional meal replacement. In the present study, we evaluated the effects of Optimized Nutri-Dense Meals on visceral fat reduction. Furthermore, as supplementary data, we evaluated outcomes with potential for improvement, such as work productivity. This intervention study may provide insights into the impact of Japan’s DRIs on visceral fat.

## 2. Materials and Methods

This study was designed and conducted following the Consolidated Standards of Reporting Trials (CONSORT) 2010 guidelines. A complete copy of the checklist is provided in Supplementary Figure S1.

### 2.1. Study Design

This four-week, open-label RCT was conducted in accordance with the principles of the Declaration of Helsinki and was approved by the Medical Station Clinic Research Ethics Committee on 14 January 2021 (approval No. 210114-3) following the ethical guidelines for research on humans (Ministry of Education, Culture, Sports, Science and Technology; Ministry of Health, Labor and Welfare, Japan). The trial was registered in the UMIN Clinical Trials Registry (registration No. UMIN000043180). Informed consent was obtained from all participants to publish the results as a treatise, under the condition that personal information would not be disclosed.

### 2.2. Study Population

Residents of Tokyo suburbs were recruited for this study through a website from 15 January 2021 to 12 February 2021. Those who wished to participate in the trial were invited to the Kouwa Clinic (Tokyo, Japan), where the details of the study and potential risks were explained, written informed consent was obtained, and the participants were screened. The inclusion criteria were as follows: (i) male individuals aged 30–64 years and (ii) body mass index (BMI) of ≥23 kg/m^2^. The participants were selected in descending order of visceral fat area. The following participants were excluded:

- Those who could not continuously consume research foods because of business trips during the intake period (those who knew in advance that their eating rate would be <80%);
- Those who were judged to be inappropriate based on the answers to the lifestyle-related questionnaire and measurements of visceral fat area and abdominal circumference;
- Those who had abnormal laboratory test values or cardiopulmonary function and were judged to have problems participating in the study;
- Those with food allergies;
- Those who would face difficulty in treatment (including dietary guidance) by participating in the trial;
- Those who used implantable medical electrical equipment such as pacemakers;
- Those who used implantable metal medical equipment;
- Those undergoing dialysis;
- Those whose physical measurements, physical tests, and clinical test values before the start of ingestion showed significant deviations from the standard range;
- Those in other clinical studies at the start of the trial;
- Those who regularly consumed foods for specified health use, foods with functional claims, health foods, and supplements that may affect test results;
- Those who regularly consumed large amounts of alcohol;
- Those who smoked extremely often (≥21 cigarettes per day);
- Those with extremely irregular diets;
- Those with irregular life rhythms, such as shift and late-night workers;
- Those who underwent health examinations two weeks before the pre-intake examination;
- Those who planned to donate blood during the research period;
- Those were judged to be inappropriate by the principal investigator.

### 2.3. Randomization

Participants were randomly assigned to two groups according to the stratified block randomization method, stratified by visceral fat area, serum triglyceride (TG) level, and calorie intake calculated using the brief self-administered diet history questionnaire (BDHQ) measured at screening. Each of these three factors was classified into two levels. For each layer generated by their combination, two groups of blocks with a block size of 4 were created by a computer (SAS 9.4 software; SAS Institute, Cary, NC, USA). The allocation manager created an allocation table, which was subsequently sealed in an envelope until the data were finalized, to ensure code breaking. The randomization process was executed by an allocation coordinator independent of the investigators.

### 2.4. Interventions

The trial was conducted in Tokyo, Japan, between 1 February and 25 May 2021. The test meals were provided in two forms: a breakfast meal set and a frozen lunch box. Participants in the test group were instructed to replace two meals, breakfast and lunch or dinner, with the test meals and to continue their usual diet with other meals. All test meals for the test groups were delivered to the participants’ homes once every two weeks in a frozen state. Moreover, the participants were advised on the optimal timing for consuming the test meals. This regimen was instructed to be followed on weekdays for four weeks.

**Figure 1:**
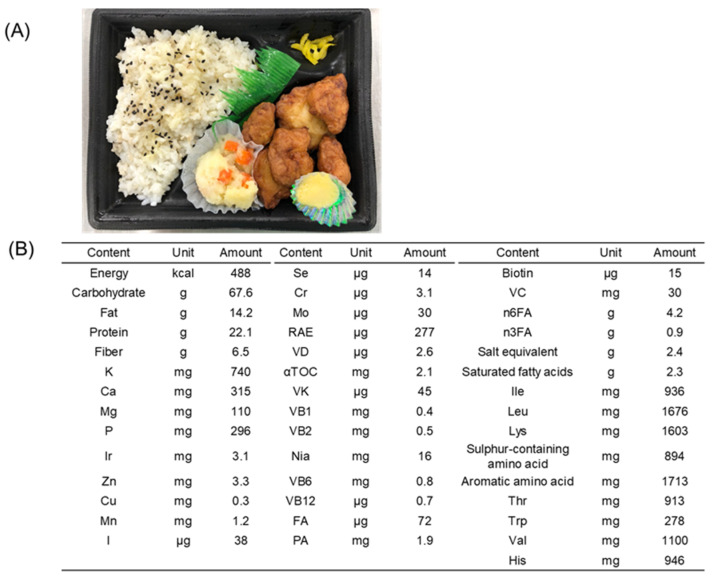
Example lunch box. (**A**) Photograph of the overall lunch box. (**B**) Nutritional components of the meal. K: Potassium, Ca: Calcium, Mg: Magnesium, P: Phosphorus, Ir: Iron, Zn: Zinc, Cu: Copper, Mn: Manganese, I: Iodine, Se: Selenium, Cr: Chromium, Mo: Molybdenum, RAE: Retinol Active Equivalent, VD: Vitamin D, αTOC: α-Tocopherol, VK: Vitamin K, VB1: Vitamin B1, VB2: Vitamin B2, Nia: Niacin, VB6: Vitamin B6, VB12: Vitamin B12, FA: Folic Acid, PA: Pantothenic Acid, VC: Vitamin C, n6FA: n6 Fatty Acid, n3FA: n3 Fatty Acid. Ile: Isoleucine, Leu: Leucine, Lys: Lysine, Thr: Threonine, Trp: Tryptophan, Val: Valine, His: Histidine.

All test meals included 33 types of nutrients within the ranges listed in Table 1. Figure 1A shows an example of a lunch box instructed for lunch or dinner. Figure 1B shows the nutrient content. All remaining menus are summarized in Supplementary Table S1. The meals could be consumed in any order from the delivered menu. Participants in the control group were instructed to consume their usual diet. No restrictions were placed on snack and alcohol consumption, but the latter was prohibited on the day before measurement in either group. Participants were asked to record the following information in a diary every day: meal record; amount of alcohol consumed; number of medicines or healthy foods, such as foods for specific health use; degree of exercise; lifestyle changes; confirmation of prohibited action; and any symptoms that were noticed. Moreover, the participants were required to wear an activity monitor (HJA-307IT; Omron Healthcare, Kyoto, Japan) that could record the total burned and active calories, and the results were recorded in a diary.

### 2.5. Outcomes

The primary outcome of this study was the change in the visceral fat area. The secondary outcomes were changes in BMI and serum TG levels. In addition, we measured the changes in gut flora through laboratory tests, and work productivity was assessed using the Japanese version of the Work Limitations Questionnaire (WLQ-J) scores. Safety was evaluated based on the number of adverse events and abnormal test results.

### 2.6. Procedures

Visceral fat area and waist circumference measurement: Visceral fat area and waist circumference were measured using an EW-FA90 (Panasonic, Osaka, Japan). This equipment measures the visceral fat area using bioelectrical impedance [16]. Before undergoing measurement, the participants were asked to eat dinner at 21:00 on the day before the test, refrain from alcohol consumption, avoid eating and drinking after 21:00 on the day before the test (participants were allowed to drink water or a small amount of warm water), avoid excessive exercise beyond the daily range the day before the test, and refrain from smoking until after the measurement. The abdomen was exposed, and a belt was worn to measure intra-abdominal resistance at the navel level.

Blood pressure measurement: Systolic and diastolic blood pressures were measured at the same time using an HEM-705IT (Omron Healthcare, Kyoto, Japan) in the morning on the measurement day. Participants sat in a chair for at least 5 min at rest after measuring their blood pressure.

Laboratory tests: The following laboratory tests were performed: total protein, total albumin, total bilirubin, direct bilirubin, alkaline phosphatase, aspartate aminotransferase, alanine aminotransferase, lactate dehydrogenase, γ-glutamyl transferase, total cholesterol, TG, high-density lipoprotein cholesterol (HDL-C), low-density lipoprotein cholesterol (LDL-C), non-HDL-C, urea nitrogen, creatinine, uric acid, sodium, potassium, chloride, glucose, and hemoglobin a1c (HbA1c).

Fecal sampling, DNA extraction, and sequencing: Fecal samples were collected using Mykinso fecal collection kits containing a guanidine thiocyanate solution (Cykinso, Inc., Tokyo, Japan) and stored at 4 °C. DNA was extracted from fecal samples using an automated DNA extraction machine (GENE PREP STAR PI-1200A; Kurabo Industries Ltd., Osaka, Japan), according to the manufacturer’s protocol. The V1–V2 region of the 16S rRNA gene was amplified using forward primers (16S_27Fmod: TCG TCG GCA GCG TCA GAT GTG TAT AAG AGA CAG AGR GTT TGA TYM TGG CTC AG) and reverse primers (16S_338R: GTC TCG TGG GCT CGG AGA TGT GTA TAA GAG ACA GTG CTG CCT CCC GTA GGA GT) with KAPA HiFi Hot Start Ready Mix (Roche). To sequence the 16S rRNA amplicons using the Illumina MiSeq platform, dual-index adapters were attached with the Nextera XT Index Kit. Each library was diluted to 5 ng/µL, and equal volumes of the libraries were mixed at 4 nM. The DNA concentration of the mixed libraries was quantified by quantitative polymerase chain reaction (qPCR) with the KAPA SYBR FAST qPCR Master Mix (KK4601, KAPA Biosystems), using primers 1 (AAT GAT ACG GCG ACC ACC) and 2 (CAA GCA GAA GAC GGC ATA CGA). Libraries were prepared according to the Illumina 16S library preparation protocol (Illumina, San Diego, CA, USA). Libraries were sequenced using the MiSeq Reagent Kit v2 (500 cycles) and 250 bp paired ends.

Taxonomy assignment based on 16S rRNA gene sequences: The paired-end reads of the partial 16S rRNA gene sequences were analyzed using QIIME 2 (version 2020.8). The steps for data processing and assignment based on the QIIME 2 pipeline were as follows: (1) DADA2 for joining paired-end reads, filtering, and denoising; (2) assigning taxonomic information to each ASV using the naive Bayes classifier in the QIIME 2 classifier with the 16S gene of the V1-V2 region data of SILVA (version 138) to determine the identity and composition of the bacterial genera.

Assessment of WLQ-J score representing presenteeism: The WLQ-J score was assessed to evaluate work-related productivity (presenteeism). The WLQ-J is a self-administered questionnaire that estimates the degree to which health problems affect job performance (work disability) and the impact of work productivity on work limitations in the previous two weeks [17]. The content of the WLQ-J questionnaire is listed in Supplementary Table S2 [18].

### 2.7. Statistical Analysis

In our previous in-house study, 31 participants with a visceral fat area of >100 cm^2^ consumed the Optimized Nutri-Dense Meals for four weeks. The Hedges effect size for the reduction in the visceral fat area was calculated to be 0.62. Furthermore, with a set alpha level of 0.05 and 80% statistical power, the sample size was determined to be 50, accounting for dropout rates, as calculated by the Bell Curve for Excel (ver. 3.23). The intervention duration in the present study was based on this previous research. The parameters analyzed in this study are presented as means ± standard deviations (SDs). The efficiency analysis was based on a per-protocol set. Safety analysis was based on a modified intent-to-treat principle (full analysis set). 

To compare the numerical data between the control and test groups, the degree of change from week 0 was assessed using Student’s *t*-test. For all two-sided tests, the significance level was set at 5%. Statistical analyses were performed using IBM SPSS Statistics version 27 (IBM Corporation, Armonk, NY, USA).

## 3. Results

### 3.1. Participants

**Figure 2:**
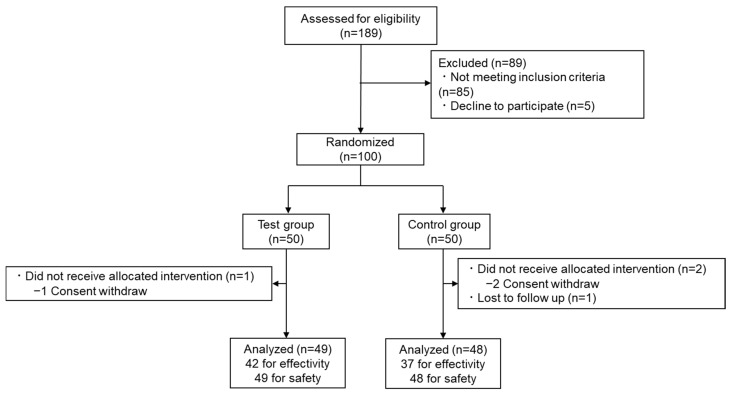
CONSORT 2010 flow diagram for study participants.

In total, 189 participants were recruited and screened, 100 of whom were enrolled and randomly allocated to the test or control groups (Figure 2). The demographic characteristics of each group are presented in Table 2. Three participants (one from the test group and two from the control group) withdrew from the study before further assessment for personal reasons unrelated to the trial. One additional participant withdrew from the study because of reasons unrelated to the trial. The overall dropout rate was 4%. Data from participants whose nutritional surveys and changes in exercise habits and lifestyles, based on interviews with the principal investigator and their diaries, were judged by the principal investigator as having the potential to interfere with the interpretation of the results and were excluded before statistical analysis. Significant differences in calorie intake calculated by BDHQ between the groups were not observed at four weeks (control, 1821 ± 594 kcal; test, 1689 ± 596 kcal; *p* = 0.330). 

### 3.2. Efficiency

The differences in the mean visceral fat area from week 0 to week 4 are shown in Table 3. Visceral fat area in the test group was more significantly reduced in the test group than that in the control group (*p* = 0.033). Moreover, BMI was more significantly reduced in the test group than that in the control group (*p* = 0.038). However, changes in TG levels were not significantly different (*p* = 0.316). Furthermore, body weight (*p* = 0.044), waist circumference (*p* = 0.033), non-HDL-C levels (*p* = 0.026), and HbA1c levels (*p* = 0.036) were more significantly reduced in the test group than those in the control group. Conversely, the occupancies of *Bifidobacterium* (*p* = 0.016) and the family *Christensenellaceae* (*p* = 0.046), as well as the WLQ-J total score (*p* = 0.020), were more significantly increased in the test group than those in the control group.

### 3.3. Safety

Eleven adverse events (four in the control group and seven in the test group) were reported (Supplementary Table S3A). The principal investigator determined that none of the adverse events were related to the test food. The values of all the parameters measured at weeks 0 and 4 to assess the safety of the intervention are shown in Supplementary Table S3B,C.

## 4. Discussion

This study showed that replacing participants’ usual diets with Optimized Nutri-Dense Meals on weekdays for four weeks reduced the visceral fat area and had additional positive effects. Visceral fat accumulation has a marked impact on CVD and metabolic syndrome [19]; thus, reducing visceral fat is important to prevent socially significant diseases. In this article, we discussed two types of interventions to combat visceral fat accumulation: nutritional education and dietary interventions. However, nutritional education is challenging in terms of exerting sufficient effects in everyday life [20]. Thus, the importance of dietary interventions, especially meal replacement, has been highlighted. The MD and washoku are potential candidates for intervention. The MD has been reported to reduce visceral fat in multiple RCTs with calorie restrictions [21,22]. In contrast, ad libitum intake of the MD did not reduce visceral fat in patients with coronary heart disease [23]. Other positive effects of the MD and washoku on visceral fat have also been reported [12,24]. However, food culture and distribution make widespread implementation of these diets difficult [14].

Here, we evaluated Optimized Nutri-Dense Meals, which is defined by nutrition content, rather than ingredients like the MD and washoku [13]. The total available energy of our intervention was relatively low (about 800 kcal/two meals on weekdays), but the diet was less strict than those in comparable studies, as other meals were not restricted in our study. Furthermore, the total calorie intake of the test group during the study period, calculated by the BDHQ, was not significantly different from that of the control group, as shown in the Results section. Therefore, the advisable adjustment of nutritional balance, rather than calorie restriction, contributed to these results.

Our study also addressed micronutrient balance. A previous cohort study revealed that seven types of micronutrients—soluble dietary fiber, manganese, potassium, magnesium, vitamin K, folic acid, and pantothenic acid—are negatively associated with visceral fat accumulation [25]. Optimized Nutri-Dense Meals may address these deficiencies (Table 1) and potentially contribute to visceral fat reduction. Although dietary fiber was enriched in Optimized Nutri-Dense Meals, additional research is required to specify the ratio of soluble-to-insoluble fiber. Furthermore, it is unclear which nutrients primarily contribute to these observed effects.

The amount of dietary saturated fatty acids (SFAs) may also contribute to visceral fat outcomes. The amount of SFAs in Optimized Nutri-Dense Meals was limited to 6.2 g per two meal portions (Table 1). A previous RCT revealed that excessive SFA intake increased visceral fat [26]. However, an epidemiological study revealed that Japanese individuals consumed a relatively high amount of SFAs, ranging from 11.6 g/day to 14.1 g/day [27]. Considering these factors, reducing SFA intake may have contributed to decreased visceral fat. 

Next, we discuss the clinical significance of primary outcome change. According to a previous systematic review, a change in the visceral fat area of 50 cm^2^ is worth a 12% change in total mortality [28]. In addition, for those with a visceral fat area of about 150 cm^2^, such as the participants in this study, reducing fat could decrease the risk of all-cause mortality [29]. Therefore, the reduction amount in our study might not be perfect but might partially contribute to risk reduction. Furthermore, we explore the reduction amount associated with other dietary interventions. Meta-analysis of probiotics resulted in a 3.6 cm^2^ reduction over 8–12 weeks of intervention [30]. Several RCTs demonstrated that the consumption of high amounts of catechins led to approximately a 6 cm^2^ decrease in visceral fat over 12 weeks [31,32]. Our dietary intervention might offer effects comparable to conventional functional foods but within a shorter duration. Optimized Nutri-Dense Meals could be considered as the primary option for further investigation.

In addition to the primary outcomes, significant improvements in health were observed across other areas, which have implications for future studies. 

Our intervention increased the occupancies of *Bifidobacterium* and the family *Christensenellaceae*. *Bifidobacterium* is known for its anti-inflammatory effects, and its levels appear to be lower in obese people than those in lean people [33]. *Bifidobacterium* is a common target for prebiotic therapies against obesity, and prebiotic oligosaccharides, such as inulin, have been shown to stimulate their growth [34]. Additionally, a fiber-rich diet has been reported to enrich *Bifidobacterium*; for example, the MD improves *Bifidobacterium* occupancy in patients with human immunodeficiency virus (HIV)-1 [35]. Because water-soluble vitamins, such as vitamins B_1_ and B_2_, are considered to be important for *Bifidobacterium* growth [36], the richness of vitamins in the diet might further contribute to their growth. The family *Christensenellaceae* has been reported to be associated with reduced weight gain [37,38]. In a study in which participants followed an MD-modified ketogenic diet for six weeks and were encouraged to consume 60–65% fats, reduce SFAs, and limit carbohydrates to 10%, the occupancy of the family *Christensenellaceae* improved [39]. Similar results were found in our study, although our novel program did not limit carbohydrate intake. The limitations of SFAs and well-balanced micronutrients may contribute to the improvement of the gut flora. Further studies are required to confirm these hypotheses.

Presenteeism, defined as going to work despite feeling unhealthy, has recently emerged as a topic of significant interest [40]. While nutritional sufficiency is associated with workplace productivity, interventions such as nutrition education have only a minor impact on presenteeism [3,41]. In our previous single-arm study, we found that our program could potentially improve presenteeism. This potential was further substantiated through our RCT.

HbA1c, representing average blood sugar levels that last several durations, also significantly improved in the test group, compared with that in the control group (Table 3). The same result was observed in a previous RCT in patients with higher HbA1c levels [42]. Nutritional content, especially fiber and vitamin D, has also been discussed. Our results indicate the robustness of this positive effect on glucose metabolism.

As mentioned in the Introduction section, this trial provides insights into the impact of dietary intervention based on the DRIs. Experts’ critical reviews created the DRIs; however, to the best of our knowledge, their effectiveness in decreasing visceral fat has not been determined [13]. Few reports of intervention studies on government-published guidelines currently exist. The food-based guidelines reported by McCarthy (U.S.’s My Plate Guide) [43] and Fuller (Australian Guideline for Healthy Eating) [44] are examples, but they are not DRIs. Since daily meals were not fully replaced in our trial, the impact of replacing all three daily meals with Optimized Nutri-Dense Meals should be determined in future studies.

To summarize, this study highlights three key strengths. First, we demonstrated the effectiveness of a dietary intervention characterized by nutrient content rather than ingredients in reducing visceral fat. This effect is likely reproducible across various cultures, provided the nutritional content aligns with our specified criteria. Notably, visceral fat is associated with an increased risk of cardiometabolic diseases, such as type 2 diabetes and hypertension [45]. Given the global prevalence of these conditions, our dietary approach could significantly aid in their prevention and management. Second, as previously mentioned, our findings support the utility of the DRI. Our intervention does not require a complete dietary overhaul, thus reducing psychological barriers for participants. Third, our study uncovered multiple potential benefits of our intervention, including improvements in presenteeism and gut flora. Presenteeism is estimated to contribute to 80% of the total costs associated with lost productivity [46]. A survey of Japanese workers suggested that economic losses attributable to presenteeism may exceed JPY 81 billion [47]. Optimized Nutri-Dense meals might serve as an innovative employee welfare strategy to combat cardiometabolic diseases and enhance workforce sustainability.

This study has five limitations. First, the intervention period was short (four weeks). As the Cochrane review on the anti-obesity effect of catechin set 12 weeks as the minimum and 12–24 months as the medium term, our program should be evaluated for such lengths in the future [48]. Thus, another trial with a longer testing period is required. Second, the number of participants was limited to obese and overweight Japanese men. Notably, visceral fat tends to decrease more in men compared with women [49]. Further studies involving women should be conducted. Because this trial focused only on Japanese populations, the effect on other ethnic groups should also be determined in the future. While the nutrient content of the test meal in this study was based on the Japanese DRIs, whether the same effect could be expected under the same conditions in other populations must be verified. Third, serum TG level, one of the secondary outcomes, did not significantly improve, although the change was sufficient. We aim to evaluate the effectiveness of TG using an appropriate sample size in future studies. Fourth, the key substances responsible for effectiveness were not isolated. Further research is required to identify the key substances and mechanisms involved. Comparisons between partially augmented dietary programs, such as vitamin- and mineral-only programs, could help solve this problem. Finally, we measured BMI or waist circumference but did not include the body shape index (ABSI) as an outcome measure. ABSI, calculated as waist circumference/(BMI^2/3^ × Height^1/2^), is distinguished from other waist circumference indices such as body roundness index and conicity index by statistical independence from BMI [50]. Future studies could enhance prediction by combining effect estimates from both ABSI and BMI.

## 5. Conclusions

Our findings indicate that Optimized Nutri-Dense Meals based on the Japanese DRIs, replacing two meals per weekday for four weeks, might have a visceral fat-reducing effect in obese and overweight men. Furthermore, BMI, waist circumference, non-HDL-C level, HbA1c level, gut flora, and presenteeism significantly improved. This study highlights the importance of continuing a dietary intervention that includes multiple nutrients to prevent visceral fat accumulation, without completely replacing the daily diet. Our results also highlight the impact of dietary patterns based on DRIs.

## Figures and Tables

**Table 1:** Limit of each nutritional component per portion of tested meals.

(A) Breakfast	/320 kcal		
		Lower limit	Upper limit
Protein	g	12.6	16.0
Fat	g	7.1	10.7
Saturated fatty acids	g		2.5
Carbohydrate	g	40.0	52.0
Fiber	g	4.1	
K	mg	484.8	
Ca	mg	155.2	262.3
Mg	mg	71.8	
P	mg	193.9	314.8
Ir	mg	2.0	5.2
Zn	mg	2.1	4.2
Cu	mg	0.2	0.7
Mn	mg	0.8	1.2
I	μg	25.2	314.8
Se	μg	5.8	47.2
Cr	μg	1.9	52.5
Mo	μg	5.8	63.0
RAE	μg	174.5	283.3
VD	μg	1.6	10.5
αTOC	mg	1.4	89.2
VK	μg	29.1	
VB1	mg	0.3	
VB2	mg	0.3	
Nia	mg	2.9	
VB6	mg	0.3	5.8
VB12	μg	0.5	
FA	μg	46.5	
PA	mg	1.2	
Biotin	μg	9.7	
VC	mg	19.4	
n6FA	g	2.1	
n3FA	g	0.4	
Salt equivalent	g		1.7
Ile	mg	264	
Leu	mg	515	
Lys	mg	396	
Sulphur-containing amino acid	mg	198	
Aromatic amino acid		330	
Thr	mg	198	
Trp	mg	53	
Val	mg	343	
His	mg	132	
(B) Lunchbox	/480 kcal		
		Lower limit	Upper limit
Protein	g	18.9	24.0
Fat	g	10.7	16.0
Saturated fatty acids	g		3.7
Carbohydrate	g	60.0	78.0
Fiber	g	6.1	
K	mg	727.3	
Ca	mg	232.7	393.4
Mg	mg	107.6	
P	mg	290.9	472.1
Ir	mg	3.1	7.9
Zn	mg	3.2	6.3
Cu	mg	0.3	1.1
Mn	mg	1.2	1.7
I	μg	37.8	472.1
Se	μg	8.7	70.8
Cr	μg	2.9	78.7
Mo	μg	8.7	94.4
RAE	μg	261.8	424.9
VD	μg	2.5	15.7
αTOC	mg	2.0	133.8
VK	μg	43.6	
VB1	mg	0.4	
VB2	mg	0.5	
Nia	mg	4.4	
VB6	mg	0.4	8.7
VB12	μg	0.7	
FA	μg	69.8	
PA	mg	1.7	
Biotin	μg	14.5	
VC	mg	29.1	
n6FA	g	3.2	
n3FA	g	0.6	
Salt equivalent	g		3.0
Ile	mg	396	
Leu	mg	773	
Lys	mg	594	
Sulphur-containing amino acid	mg	297	
Aromatic amino acid		495	
Thr	mg	297	
Trp	mg	79	
Val	mg	515	
His	mg	198	

K: Potassium, Ca: Calcium, Mg: Magnesium, P: Phosphorus, Ir: Iron, Zn: Zinc, Cu: Cupper, Mn: Manganese, I: Iodine, Se: Selenium, Cr: Chromium, Mo: Molybdenum, RAE: Retinol active equivalent, VD: Vitamin D, αTOC: α-tocopherol, VK: Vitamin K, VB1: Vitamin B1, VB2: Vitamin B2, Nia: Niacin, VB6: Vitamin B6, VB12: Vitamin B12, FA: Folic acid, PA: Pantothenic acid, VC: Vitamin C, n6FA: n6 Fatty acid, n3FA: n3 Fatty acid, Ile: Isoleucine, Leu: Leucine, Lys: Lysine, Thr: Threonine, Trp: Tryptophan, Val: Valine, His: Histidine.

**Table 2:** Participant characteristics.

	Unit	Control	Test	***p***
Age	years	49.5 ± 9.3	48.2 ± 10.0	0.524
Body weight	kg	82.1 ± 8.6	81.0 ± 7.5	0.502
BMI	kg/m^2^	27.9 ± 2.9	27.8 ± 2.1	0.856
SBP	mmHg	125.9 ± 13.3	125.5 ± 11.1	0.870
DBP	mmHg	79.3 ± 10.0	79.6 ± 11.4	0.876
Visceral fat area	cm^2^	162.0 ± 26.6	163.0 ± 28.3	0.855
Waist circumference	cm	98.1 ± 6.2	98.6 ± 6.1	0.707
TG	mg/dL	157.9 ± 78.7	158.1 ± 76.6	0.990
HDL-C	mg/dL	50.4 ± 11.0	57.0 ± 14.0	0.011
LDL-C	mg/dL	140.6 ± 29.6	142.4 ± 38.9	0.794
Non-HDL-C	mg/dL	174.1 ± 35.4	174.9 ± 45.0	0.917
Glu	mg/dL	91.1 ± 7.5	91.3 ± 7.3	0.850
HbA1c	%	5.4 ± 0.3	5.4 ± 0.3	0.421

Each value is expressed as the mean ± SD. BMI, body mass index; SBP, systolic blood pressure; DBP, diastolic blood pressure; TG, triglyceride; LDL-C, low-density lipoprotein cholesterol; HDL-C, high-density lipoprotein cholesterol; Glu, fasting blood glucose; HbA1c, hemoglobin A1c; SD, standard deviation.

**Table 3:** Evaluation of the effectiveness of the trial.

	Unit		n	0 Week	4 Week	⊿	*p*
						95%CI	
Visceral fat area	cm^2^	Control	37	152.5 ±31.4	158.1 ± 28.9	−7.5	0.033
(Primary outcome)		Test	42	161.0 ± 31.0	159.1 ± 33.6	(−14.3 to −0.6)	
BMI	kg/m^2^	Control	37	27.5 ± 3.0	27.4 ± 3.0	−0.2	0.038
(Secondary outcome)		Test	42	27.6 ± 2.2	27.3 ± 2.2	(−0.3 to −0.01)	
TG	mg/dL	Control	37	153.9 ± 71.8	152.6 ± 69.5	−11.6	0.316
(Secondary outcome)		Test	42	150.6 ± 76.3	137.7 ± 71.9	(−34.4 to 11.2)	
Body weight	kg	Control	37	80.4 ± 8.0	80.1 ± 7.9	−0.5	0.044
		Test	42	80.1 ± 7.9	79.4 ± 8.1	(−1.0 to −0.001)	
Waist circumference	cm	Control	37	97.2 ± 5.9	97.5 ± 5.8	−1.1	0.033
		Test	42	98.8 ± 6.0	98.0 ± 6.1	(−2.0 to −0.1)	
HDL-C	mg/dL	Control	37	51.2 ± 11.6	51.0 ± 12.5	0.3	0.847
		Test	42	57.1 ± 13.6	57.2 ± 13.7	(−2.4 to 2.9)	
LDL-C	mg/dL	Control	37	142.5 ± 34.8	145.6 ± 32.0	−8.1	0.078
		Test	42	148.1 ± 37.5	143.1 ± 45.0	(−17.2 to 0.9)	
Non-HDL-C	mg/dL	Control	37	174.1 ± 39.7	176.4 ± 38.2	−10.8	0.026
		Test	42	179.1 ± 44.4	170.6 ± 52.5	(−20.3 to −1.3)	
Glu	mg/dL	Control	37	90.4 ± 6.8	89.3 ± 7.1	0.4	0.698
		Test	42	90.5 ± 6.6	89.8 ± 6.2	(−2.7 to 1.8)	
HbA1c	%	Control	37	5.4 ± 0.3	5.5 ± 0.3	−0.1	0.036
		Test	42	5.4 ± 0.0	5.4 ± 0.3	(−0.1 to −0.004)	
Occupancy of	%	Control	37	3.4 ± 3.6	2.8 ± 2.9	1.5	0.016
*Bifidobacterium*		Test	42	3.2 ± 4.4	4.1 ± 3.2	(0.3 to 2.7)	
Occupancy of the family	%	Control	37	0.3 ± 0.8	0.2 ± 0.5	0.4	0.046
*Christensenellaceae*		Test	42	0.2 ± 0.6	0.5 ± 1.6	(0.01 to 0.8)	
WLQ-J	points	Control	32	98.1 ± 2.7	97.4 ± 3.0	1.2	0.020
		Test	36	97.9 ± 2.3	98.3 ± 1.9	(0.2 to 2.3)	

Each value is expressed as the mean ± SD. BMI, body mass index; TG, triglyceride; LDL-C, low-density lipoprotein cholesterol; HDL-C, high-density lipoprotein cholesterol; Glu, fasting blood glucose; HbA1c, hemoglobin A1c; WLQ-J, Work Limitation Questionnaire Japanese; SD, standard deviation.

## Data Availability

Data would be provided to the researchers who presented plans with appropriate methodologies and obtained consent from all co-authors.

## Supplementary Materials

The following supporting information can be downloaded from https://www.mdpi.com/article/10.3390/nu16183202/s1: Figure S1, CONSORT2010 checklist; Table S1, Complete menu of test meals; Table S2, Questions of WLQ-J; Table S3A, All adverse events reported throughout the study; Table S3B, Blood test results; Table S3C, Urinalysis results.
